# Conserved G-Quadruplex Motifs in Gene Promoter Region Reveals a Novel Therapeutic Approach to Target Multi-Drug Resistance *Klebsiella pneumoniae*

**DOI:** 10.3389/fmicb.2020.01269

**Published:** 2020-06-26

**Authors:** Uma Shankar, Neha Jain, Subodh Kumar Mishra, Tarun Kumar Sharma, Amit Kumar

**Affiliations:** ^1^Discipline of Biosciences and Biomedical Engineering, Indian Institute of Technology Indore, Indore, India; ^2^Translational Health Science and Technology Institute, Faridabad, India

**Keywords:** g-quadruplex, therapeutic target, *Klebsiella pneumoniae*, non-canonical structures, multi-drug resistance

## Abstract

An opportunistic pathogen, *Klebsiella pneumoniae* is known to cause life-threating nosocomial infection with a high rate of morbidity and mortality. Evolutions of multi-drug-resistant and hyper-virulent strains of *K. pneumoniae* make the situation worse. Currently, there is no incisive drug molecule available for drug-resistant hyper-virulent *K. pneumoniae* infection that emphasizes the need for identification of novel and more promising drug targets in *K. pneumoniae*. Recently, various non-canonical structures of nucleic acids especially G-quadruplex (G4) motifs have been identified as potential therapeutic targets against several human pathogenic bacteria and viruses including *Mycobacterium tuberculosis*, *Streptococcus pneumoniae*, human immunodeficiency virus (HIV), Ebola, and Nipah. Therefore, in present study we screened the *K. pneumoniae* genomes for identification of evolutionary conserved G4 structure-forming motifs as promising anti-bacterial drug targets. Bioinformatics analysis revealed the presence of six highly conserved G4 motifs in the promoter region of five essential genes that play a critical role in nutrient transport and metabolism. Biophysical studies showed the formation of G4 structure by these conserved motifs. Circular Dichroism melting analysis showed the stabilization of these G4 motifs by a well-known G4-stabilizing agent, BRACO-19. The stabilization of these motifs by BRACO-19 was also able to stop the primer extension process, which is an essential phenomenon for expression of the G4-harboring gene. The addition of G4-specific ligand at low micromolar range was observed to be lethal for the growth of this bacteria and negatively controlled the expression of the G4-harboring genes via G4 structure stabilization. These observations strengthen the formation of G4 structures by the predicted G4 motif *in vivo*, which can be stabilized by G4 ligands like BRACO-19. This stabilization of G4 structures can attenuate the expression of G4-harboring essential genes and thus play a critical role in the regulation of gene expression. Thus, taking all given result in consideration, for the first time, this study showed the new therapeutic avenue for combating *K. pneumoniae* infection by characterizing the conserved G4 motifs as promising therapeutic targets.

## Introduction

*Klebsiella pneumoniae*, a Gram-negative bacteria, causes ample number of life-threating diseases, including pneumonia, urinary tract infection, cystitis, endocarditis, sepsis, and blood stream infections and are the leading source of hospital acquired (nosocomial) infections (Paczosa and Mecsas, 2016). They are also the leading cause of the worst community-acquired infections like pyogenic liver abscesses, endogenous endophthalmitis, and necrotizing pneumoniae (Sánchez-López et al., 2019). The main reservoirs of *K. pneumoniae* are the gastrointestinal tract and medical personnel and equipment (Podschun and Ullmann, 1998). Though *K. pneumoniae*-preferred hosts are immunocompromised individuals, the emergence of multi-drug resistant (MDR), extensively drug resistant (XDR), and hyper-virulent strains make healthy individuals susceptible to infection also. This makes *K. pneumoniae* among one of the most concerning pathogens, classified under ESKAPE organisms that currently have no potential treatment available (Boucher et al., 2009; Bassetti et al., 2018; Russo and Marr, 2019). Various antibiotics like colistin, fosfomycin, and aminoglycosides are used in combination, but provide unsatisfactory results (Bassetti et al., 2018); as a consequence, they are associated with high morbidity and mortality. Infection of MDR and XDR strains of *K. pneumoniae* in critically immunosuppressed patients and organ transplant recipients has a mortality rate greater than 50%, making it one of the major challenges to the clinicians (Xu et al., 2017). Henceforth, identification of novel targets and a better understanding of *K. pneumoniae* physiology that helps the bacterium to survive and persist in the host will lead to the development of a new strategies that can be used for combating this deadly pathogenic infection.

Understanding the molecular mechanisms of gene expression provides a better insight into its regulation. Recent studies have shown that gene expressions are regulated at various stages, including the transcription and translation levels, and thus, complete or partial suppression of essential genes hinders the growth of pathogenic bacterium, leading to its decreased growth and virulence. In the last decade, identification of various non-canonical secondary structures of nucleic acid have shown an additional layer of gene expression regulation and are being used as potential therapeutic targets. G-quadruplexes (G4s) are one of the highly studied secondary structures of these nucleic acids that are present in the G-rich region of the genome. They are reported to form a diverse array of stable topologies and can be intramolecular with a single strand participating or intermolecular where two, three, or four different strands participate in the G4 formation (Rhodes and Lipps, 2015; Supplementary Figure S1). The G4 stability and diversity are further influenced by the number and the length of G-tracts; spacer length and composition; strand orientation (parallel, antiparallel, or mixed/hybrid); and presence of bulges, ligands, or physiological conditions (Hazel et al., 2004; Burge et al., 2006). Under physiological conditions, the stable G4 structure leads to knot formation in the genome, obstructing replication, transcriptional, and translational machinery, thereby playing a major role in gene expression regulation. G4s are abundantly reported in promoters and UTR, which strengthens their role as transcriptional and translational regulators (Rhodes and Lipps, 2015), and in the telomeric regions, where they regulate cell division and aging (Tawani and Kumar, 2015). Due to their role in gene regulation, ample number of research have shown these non-canonical G4 structures as effective anti-cancer, anti-bacterial, and anti-viral therapeutic targets (Buket et al., 2014; Ruggiero and Richter, 2018; Asamitsu et al., 2019; Saranathan and Vivekanandan, 2019; Brazda et al., 2020a). Recent analysis of genome mining in bacterial domains revealed non-random distribution of G4 motifs in their genome, with the highest frequency in the harsh environment-resistant Deinococcus-Thermus phylum, while the lowest was in Thermotogae (Bartas et al., 2019). The presence of G4 motifs in the extremophiles like *Deinococcus radiodurans* and *Thermus aquaticus* might help in surviving the extreme environment (Kota et al., 2015; Ding et al., 2018). Various other studies have shown the presence of evolutionarily conserved G4 motifs in the crucial genomic locations in several bacteria and protozoans, including *Escherichia coli* (Kaplan et al., 2016), *Neisseria gonorrhea* (Cahoon and Seifert, 2009), *M. tuberculosis* (Perrone et al., 2017; Mishra et al., 2019b), *S. pneumoniae* (Mishra et al., 2019a), and *Plasmodium falciparum* (Harris et al., 2018). The G4 binders TMPyP4, ex-NDI 2, and BRACO-19 are reported to inhibit bacterial growth and virulence via a G4-mediated mechanism (Perrone et al., 2017; Mishra et al., 2019a, b). Conserved G-rich regions in the Zika virus (Fleming et al., 2016), Herpes Simplex virus (Artusi et al., 2015), Nipah (Majee et al., 2020a), Adenovirus (Majee et al., 2020b), and other viruses have also been shown to form G4 conformations and are proposed as anti-viral drug targets (Ruggiero and Richter, 2018).

These studies summarize the potential of G4s as a drug target for anti-bacterial and anti-viral therapeutic targets. Though several bacterial genomes are reported to harbor G4 sequences, to the best of our knowledge, no systematic analysis for the screening and characterization of G4 motifs has been done for *K. pneumoniae*, so far. Therefore, in the present work, we sought to explore the *K. pneumoniae* genome for the conserved G4 motifs that can be used as promising therapeutic targets in this notorious pathogen using our previously developed G4 prediction tool (Mishra et al., 2016) and their genomic location [promoter/upstream region or open reading frames (ORFs)] was annotated manually. Intriguingly, the identified conserved G4 motifs were found to be present in both drug-sensitive and drug-resistant strains of the bacterium, which makes them universal targets for treatment of *K. pneumoniae* infection. G4 motifs, when present in the ORFs, have been shown to affect the gene expression by halting the translational machinery, while the G4 motifs present at the upstream, and/or promoter regions have been shown to affect the expression of the gene by inhibiting both transcription and translation. Due to their regulatory role at both the transcription and translational level, the G4 motifs present at the promoter regions have been widely investigated. For example, the G4 motif present at the upstream of *pilE* gene mediates antigenic variation in *N. gonorrheae* (Cahoon and Seifert, 2009), while motifs present at the upstream/promoter region of *M. tuberculosis* genes inhibits their expression (Perrone et al., 2017). Gamma radiation-resistant genes of *D. radiodurans* are differentially regulated by the G4 motifs present in their promoter region (Kota et al., 2015). Similarly, G4 motifs present in the promoter regions of *nas* genes in *Paracoccus denitrificans* negatively regulate the nitrate assimilation in the bacterium (Waller et al., 2016). In human immunodeficiency virus (HIV), G4 at the promoter region of a unique long terminal repeat is being studied as a potential anti-HIV therapeutic targets (De Nicola et al., 2016). Similarly, in Hepatitis B virus, G4 in the promoter region of the *preS2/S* gene upon stabilization affects its expression (Biswas et al., 2017). Thus, we focused on the motifs present at the upstream/promoter regions of the genes and selected six sequences having the propensity to form G4 structure. We employed the NMR and Circular Dichroism (CD) spectroscopy to characterize the formation of G4 structure by predicted conserved G4 motifs. We also studied the interaction of these G4 structures with BRACO-19, a well know G4 ligand using isothermal calorimetry assay and primer extension assay. Further, we also tested the differential expression of the G4-harboring gene in the presence of BRACO-19 using quantitative RT-PCR assay. At physiological conditions, all the six selected G4 motif sequences were able to form stable G4, *in vitro*. BRACO-19 specifically interacted with the G4s and negatively affected the expression of genes harboring the G4 motifs in their promoter region. Overall, this study supports the plausible biological role of G4s in *K. pneumoniae* that can be used as therapeutic drug targets against this pathogen.

## Materials and Methods

### Prediction of Putative G4 Forming Sequences and Their Functional Annotation in *Klebsiella pneumonia* Genome

The complete genome sequences of the *Klebsiella pneumonia* were retrieved from NCBI Genome Database^1^. KP-PGQs (*Klebsiella pneumoniae* putative G-quadruplex sequences) were predicted by previously developed G4 prediction tool (Mishra et al., 2016). The tool is based upon the algorithm that uses the following equation to explore the PGQs in *Klebsiella pneumonia*:

$$G_{\left(3+\right)}{\left[N\right]}_{\left(1-7\right)}G_{\left(3+\right)}{\left[N\right]}_{\left(1-7\right)}G_{\left(3+\right)}{\left[N\right]}_{\left(1-7\right)}G_{\left(3+\right)}$$

Where **G** refers to Guanine and **N** refers to any nucleotide including Guanine.

Both positive and negative strands of the genome were used for G4 prediction. To find the conserveness of the KP-PGQs in the *Klebseilla pneumonia* genus, manual clustering was performed based on the G4 motif sequences, and the frequency of occurrence was calculated. Further, to functionally annotate the KP-PGQs, the predicted motif genomic locations were analyzed using NCBI Genbank graphic mode. All the conserved KP-PGQs, along with 5 bps upstream and downstream, were retrieved for all the completely sequenced strains, sequentially aligned by using Clustal Omega tool and consensus logo were generated by using WebLogo 2 tool (Crooks et al., 2004).

### Oligonucleotide Sample Preparations

*Klebsiella pneumoniae* putative G-quadruplex sequences Oligonucleotides were purchased from Sigma Aldrich (Bangalore, India), and 100 μM stock solutions were prepared in MiliQ water according to manufacturer’s protocol and stored at −20°C (Supplementary Table S1). For CD and electrophoretic mobility shift assay (EMSA) experiments, the stock solutions were diluted to 20 μM in Tris–HCl buffer (100 mM with pH 7.4), having 50 mM of K^+^, Na^+^, or Li^+^. The diluted samples were denatured by heating at 92°C for 10 min and kept overnight for reannealing at room temperature.

### NMR Spectroscopy

For 1D ^1^H NMR experiments, an NMR Spectrometer [Model AVANCE III 400 Ascend Bruker BioSpin International AG, Switzerland equipped with 5-mm broadband inverse (BBI) probe] was used. The lyophilized oligonucleotide samples were dissolved in deionized water and denatured at 92°C for 10 min. Thereafter, 100 mM potassium phosphate buffer (pH 7.2) containing 50 mM KCl and 10% deuterated water (D_2_O) was added, making the oligonucleotide’s final concentration of 200 μM. 3-(Trimethylsilyl) propionic-2, 2, 3, 3-D4 acid sodium salt (TSP) was used as an internal reference. NMR spectra were recorded at 298 K with 20-ppm spectral width and were analyzed using Bruker Topspin software (Version 1.3).

### Circular Dichroism Spectra and Melting Analysis

Circular dichroism spectroscopy was performed using Jasco J-815 CD spectrometer (Jasco Hachioji, Tokyo, Japan) attached with a PTC-423S/15 Peltier Temperature Controller by using a Quartz cuvette of 1 mm optical length and a sample volume of 200 μl. To prevent the condensation around the cuvette as a result of heating, nitrogen gas was supplied continuously. The oligonucleotide solutions with a final concentration of 20 μM were dissolved in water or Tris–HCl buffer containing 50 mM K^+^, Na^+^, or Li^+^ and incubated overnight after denaturation at 92°C for 10 min. Spectra were analyzed at 25°C within the wavelength range of 220–320 nm and a scanning speed of 20 nm/min. Spectral accumulations were recorded in triplicate, and average absorbance was taken for the analysis. To nullify the contribution of the respective buffers, before each spectra analysis, baseline correction was performed by using just the buffer in the cuvette. For CD melting analysis, the data were collected at the wavelength that provided the highest ellipticity in the CD spectra for KP-PGQs in the respective buffer within the temperature range of 25 to 98°C, with a rising rate of 1°C/min. Also, CD melting analysis was performed in the presence and absence of BRACO-19 with Drug/Nucleotide (D/N) ratio of 0 and 1 and the change in melting temperature (Δ*T*_*m*_) was observed. The resultant spectra and melting data were smoothened and analyzed using Sigma plot 12.5.

### Electrophoretic Mobility Shift Assay

To analyze the shift in mobility of KP-PGQs in the absence or presence of various cations, non-denaturing native polyacrylamide gel (20%) was run in 1 × Tris–Boric acid–EDTA buffer at 4°C in a vertical electrophoresis unit (Bio-Rad Mini protean Tetra) at a constant voltage of 90 V. Each KP-PGQ was dissolved in either in the absence of any cation or in the presence of 100 mM K^+^, Na^+^, or Li^+^ at a final oligonucleotide concentration of 20 μM. For comparative analysis of the mobility shift between G4 sequences and non-G4 sequences, a mutant of similar length as that of KP-PGQs were constructed by replacing the guanines involved in G4 formation with thymine and analyzed using G4 Killer tool (available at^2^). The target G4 hunter score threshold was kept as 1.2, similar to that used in G4Hunter tool for G4 prediction (Brazda et al., 2020b; Supplementary Table S2). A well-known G4 forming motif, *ckit21*, and its mutant dissolved in K^+^ buffer were taken as a positive control. The gels were stained using Ethidium bromide for 10 min and visualized under ImageQuant LAS4000 (GE Healthcare, United States).

### KP-PGQs Interaction Analysis With BRACO-19

To check the interaction of KP-PGQs with BRACO-19, isothermal calorimetry (ITC; MicroCal iTC200, GE Healthcare, United States) was utilized. 16 μM KP-PGQ and 50 μM BRACO-19 (stock solution) dissolved in 1 × potassium phosphate buffer (pH = 7.2) were used for analysis. BRACO-19 was added to the oligonucleotides by a total of 21 injections—1.78 μl each with the duration of 3.5 s each. Spacing between subsequent injections was kept to 90 s each. Heat of dilution was observed by injecting a similar concentration of BRACO-19 into 1 × potassium buffer and subsequently was subtracted from the binding isotherm of the KP-PGQs. The resultant isotherms were fitted using a two-site binding model, and thermodynamic parameters were analyzed using MicroCal Origin version 7 software. Thermodynamic feasibility was analyzed by observing the changes in Gibbs free energy (ΔG).

### PCR Primer Extension Assay

The KP-PGQ templates, a linear template (control), their reverse complimentary primers, (Supplementary Table S3), and *Taq* polymerase were purchased from Sigma Aldrich. Six 25 μl reaction mixtures were prepared consisting of 1 × PCR Buffer, 4 mM MgCl_2_, 2 nM of each template, and its respective reverse complimentary primer, 0.33 mM dNTPs, 50 mM of KCl, and 2.5 units of *Taq* polymerase. Various concentrations of BRACO-19 ranging from 0–4 μM were added in each reaction mixture, and the reactions were amplified in a Master cycler Nexus gradient (HiMedia). The PCR cycle was initiated with an initial denaturation at 95°C for 2 min, followed by 25 cycles of denaturation at 95°C for 30 s, annealing at 58°C for 30 s, and extension at 48°C for 1 min. A final extension at 48°C for 10 min. The products were then run on a 3% agarose gel pre-stained with EtBr, and the band intensities were analyzed by using ImageQuant LAS 4000.

### *Klebsiella pneumoniae* Growth Inhibition Analysis

To evaluate minimum inhibitory concentration of BRACO-19 for *K. pneumoniae*, MTT-based growth inhibition assay was performed, as per the modified protocol mentioned elsewhere (Kerbauy et al., 2016; Lalitha et al., 2017). Briefly, the *K. pneumoniae* ATCC 700603 strain procured from HiMedia was revived and cultured in BHI broth media (HiMedia) till it reached an optical density of 0.5 at 600 nm (O.D._600_). The cultures were then diluted 100 times using fresh BHI media and 200 μl was transferred in each well of a 96-well plate. BRACO-19 dilutions were prepared in the BHI media from the freshly prepared solution of 80 mM and were added in the respective wells; the last well served as blank. The experiments were performed in triplets. The plates were kept at 37°C for 24 h and thereafter 20 μl of MTT (stock solution of 5 mg/ml) was added and incubated further for 3 h. Afterward, the formazan precipitate was diluted using 100 μl DMSO and color intensities were observed under microplate reader (BioTek) at 570 nm. If overflow occurred, it was further diluted using equal amount of DMSO in all wells.

### RNA Isolation and RT-PCR Assay

*Klebsiella puemoniae* ATCC 700603 was first cultured in three culture tubes to O.D._600_ = 0.5; two of these tubes were then treated with 5.38 μM and 10.77 μM BRACO-19 for 80 min at 220 rpm and 37°C while the third one served as untreated/control. The cells were then pellet down by centrifugation at 5,000 rpm for 10 min at 4°C and re-suspended in RNA protect reagent (Qaigen, United States). TRIZOL reagent (Invitrogen, Carlsbad, California, United States) was used for RNA isolation as per the manufacturer’s instructions. The purity (A260/280) and concentration (in ng/μl) of the isolated RNA was checked by using Nanodrop (Thermo scientific, Waltham, Massachusetts, United States).

RT-PCR was performed using DNAase-treated RNA samples of treated and non-treated condition, as described earlier (Mishra et al., 2019b). Briefly, cDNA was synthesized in a 20 μl reaction using iScript^TM^ cDNA synthesis kit, and RT-PCR reaction was carried out using iTaq^TM^ Universal SYBR^®^ Green qPCR master mix, with 0.5 μM of forward and reverse primers (Supplementary Table S4) and 2 μl of the respective cDNA in a total reaction volume of 25 μl in a 96-well RT-PCR plate in Step One Plus Thermal Cycler (Applied Biosystem, United States). Thermocycler conditions were as follows: initial denaturation at 92°C for 2 min, 30 cycles of 92°C for 20 s, and 53°C for 1 min and a final extension at 53°C for 10 min. All the reactions were performed in three biological replicates and the data were analyzed by using comparative Ct method (ΔΔCt) where Ct values of each sample were normalized with respect to (w.r.t.) the culture control. The expression of 16S rRNA was used as a house keeping reference.

## Results

### Motif Analysis Revealed the Presence of G4s in the *Klebsiella pneumoniae* Genome

**FIGURE 1:**
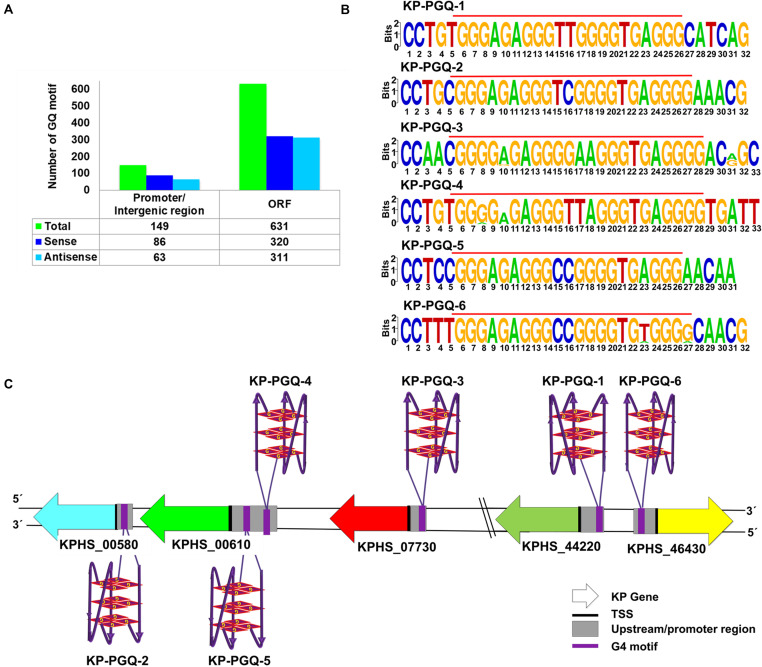
G-quadruplex motifs in *Klebsiella pneumoniae.* **(A)** Bar graph depicting the number of G4 motifs in promoter/intergenic region and ORF for *Klebsiella pneumoniae* subsp. *pneumoniae* HS11286. **(B)** Sequence logo of the highly conserved six G4 motifs present in the promoter region of *Klebsiella pneumoniae*. **(C)** Schematic representation of six G4 motifs at the promoter regions of the five genes in the *Klebsiella pneumoniae* subsp. *pneumoniae* HS11286 genome along with the strand information in which these PGQs are located.

Guanine-rich regions present in the nucleic acids tend to form G4s*in vitro* and *in vivo* and act as regulatory elementsin various biological phenomena. Four consecutive tracts having aminimum of two guanines separated with a spacer region are requisite for the formation of G4 (Ruggiero and Richter, 2018). Though G-tract with two guanines can form G4 conformation, it’s widely reported that tracts having three or more guanines provide additional stability to G4 *in vivo*. With this consideration, we screened 387 completely sequenced strains of *K. pneumoniae* available at NCBI until October 2019 (Supplementary Dataset File 2) to identify putative G-quadruplex motifs (KP-PGQs) by using our previously developed G4 prediction tool (Mishra et al., 2016). *K. pneumoniae* have a genomic length of ∼5.682 Mb and a GC content of ∼58%. As in bacteria, transcription is bidirectional, and both strands participate in gene expression; therefore, both the strands were searched for the PGQs motifs. Bioinformatics analysis revealed ∼770 motifs having the potential to form G4 with a threshold of G-tract ≥ 3 and spacer length ≥ 1 and ≤7 (Supplementary Dataset File 2). For the reference strain *K. pneumoniae* HS11286, total 780 G4 motifs were obtained, out of which 406 PGQ motifs were in the sense and 374 motifs in the antisense strand, with a G4 density (PGQs per kb) of 0.071 for sense and 0.065 in the antisense strand (Supplementary Dataset File 2). On functional annotation, 149 PGQs (86 in sense and 63 in antisense) were found to be located in the promoter and/or intergenic regions, and 631 motifs (320 in sense and 311 in antisense strand) were located in the ORFs (Figure 1A). The presence of highly conserved motifs in the genomes depicts their critical role in the organism’s survival; therefore, the predicted PGQs were clustered based on the sequence similarity, and the frequency of occurrence in the *Klebsiella* genus was observed. The PGQs depicting ≥90% frequency of occurrence in the *K. pneumoniae* genus are enlisted in Table 1 and Supplementary Table S5. Interestingly, genes harboring these conserved PGQs were the part of various essential pathways of *K. pneumoniae* that plays a potential role in the bacterial survival. The KP-PGQ G4 motifs along with 5-bps upstream and downstream residues were used for constructing the consensus logo of the KP-PGQs. Upon analyzing the consensus logo, conserved-ness in both the G-tract and spacer sequence was observed (Figure 1B and Supplementary Figure S2). The propensity of G4 formation by the conserved motifs was further confirmed by G4Hunter and QGRS Mapper tool (Supplementary Tables S6, S7). Various *in silico* studies have shown a high frequency of G4 motifs near the promoter regions in both eukaryotic and prokaryotic genomes (Rawal et al., 2006; Huppert and Balasubramanian, 2007). Due to their dynamic behavior, G4s at the upstream of the transcription start sites influence the gene expression to a great extent by acting as *cis*-regulating elements at the transcription and translation level (Perrone et al., 2013; Fedeles, 2017; Perrone et al., 2017). Therefore, among the highly conserved KP-PGQs, the motifs located at the promoter/upstream regions of the genes were selected for further analysis (Figure 1C). Functional annotation revealed the location of KP-PGQ-1 at the 5’ upstream of D-erythrose 4-phosphate dehydrogenase (KPHS_44220). KP-PGQ-2 and KP-PGQ-3 were located at the upstream of alcohol dehydrogenase (KPHS_00580) and L-ribulose-5-phosphate 4-epimerase (KPHS_07730), respectively. Interestingly, KP-PGQ-4 (117 bp upstream, in sense strand) and KP-PGQ-5 (42 bp upstream, in antisense strand) were present at the upstream of an ABC transporter (KPHS_00610). Likewise, KP-PGQ-6 was located at the upstream of 2,4-dienoyl-CoA reductase (KPHS_46430). The detailed information regarding the location of G4s, its strand, gene associated with the G4, and its directions along with the conserved frequencies are indicated in Table 1. As KP-PGQ-3, KP-PGQ-5, and KP-PGQ-6 were present on the sense strand *w.r.t.* gene orientation, their stabilization may lead to the gene expression regulation at a translational level while rest three PGQs present at the antisense strand may act as regulators at the transcriptional level (Agarwal et al., 2014). These six selected highly conserved G4 motifs located at the upstream location of KP genes were analyzed by various biophysical and biomolecular techniques for the formation of G4, *in vitro*. As KP-PGQ-3, KP-PGQ-5, and KP-PGQ-6 were present on the sense strand *w.r.t.* gene orientation, their stabilization may lead to the gene expression regulation at a translational level, while rest three KP-PGQs present at the antisense strand may act as regulators at the transcriptional level (Agarwal et al., 2014).

**TABLE 1:**
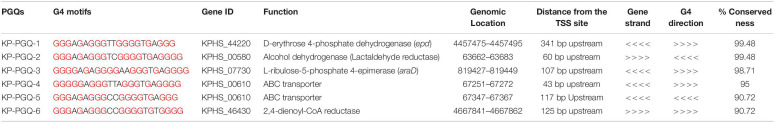
List of Conserved KP-PGQs present at the upstream/promoter region of genes in *Klebsiella pneumoniae* genome with their location and functional annotation.

*(>>>> sense strand, <<<< Antisense Strand, TSS, transcription start site; ORF, open reading frame). The red letters are Guanines in the DNA sequences that interacts to form G-quadrets.*

### 1D ^1^H NMR Spectral Analysis Affirmed the Formations of G4 Structures in the Highly Conserved KP-PGQs

**FIGURE 2:**
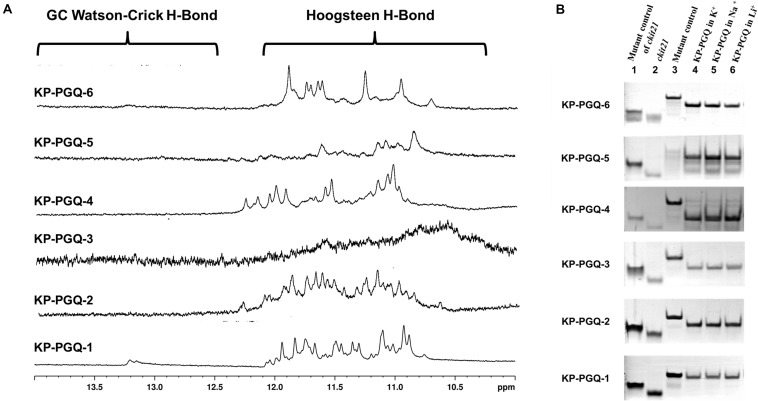
NMR and EMSA analysis. **(A)** NMR spectra (from 10–14 ppm) of six conserved G4 sequences obtained in the presence of K^+^ cation. All the six KP-PGQs showed the chemical shift in the range of 10–12 ppm affirming the formation of G4. **(B)** Electrophoretic mobility shift assay of KP-PGQs depicting the formation of intramolecular G4 in the conserved KP-PGQ sequences.

Various standard spectroscopic techniques, including NMR and CD, were exploited to confirm the formation of G4s in the predicted PGQ sequences. One-dimensional proton nuclear magnetic resonance spectroscopy (1D ^1^H NMR) gives a glimpse of the presence of canonical and non-canonical hydrogen bonds in nucleic acids and is considered a classical technique for the analysis of G4s. A chemical shift in the spectral range of 10–12 ppm depicts the formation of Hoogsteen hydrogen bonds formed between guanine imino-protons in G4s, while the chemical shift due to the formation of canonical hydrogen bonds in Watson–Crick base pairing comes in the range of 12–14 ppm (Feigon et al., 1995; Adrian et al., 2012). Therefore, 1D ^1^H NMR was performed for the predicted conserved G4s sequences in the presence of K^+^ ion. All the six PGQs displayed the chemical shift in the range of 10–12 ppm with clear peaks except KP-PGQ-3 where the shift was observed, but the peaks were not clearly visible. The presence of chemical shift at 10–12 ppm affirmed the formation of Hoogsteen hydrogen bonds, thereby confirming the presence of G4 conformation in these KP-PGQs (Figure 2A).

### EMSA Asserts the Formation of Intramolecular G4 Conformation in the Predicted KP-PGQs

Formation of secondary structures like G4s, hairpins, etc. leads to increase in mobility due to compact topology, as compared to that of a linear chain of similar lengths. Similarly, due to the difference in molecular weight, intramolecular G4 moves faster as compared to that of an intermolecular molecule. This change in mobility can be analyzed using EMSA, where the molecules are run on a non-denaturing polyacrylamide gel, and shifts in the bands are visualized. EMSA was performed for all six KP-PGQs in the presence of three cations, namely K^+^, Na^+^, and Li^+^. In the presence of all three cations, KP-PGQs were observed to form a single band and migrated faster in the presence of all three cations as compared to their mutant sequence (Figure 2B). This observation confirmed the formation of intramolecular G4 conformations in the KP-PGQs sequences. *ckit21* G4 sequence used as a positive control also moved faster as compared its mutant depicting the formation of intramolecular G4 conformation (Figure 2B).

### Presence of Mono-Cations Affects the Topology of the Conserved KP-PGQs

**FIGURE 3:**
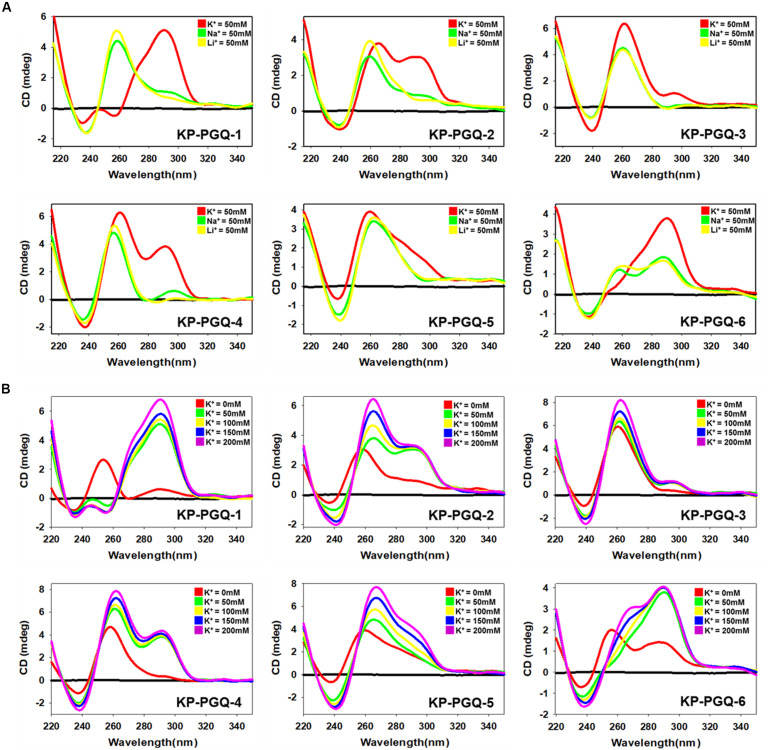
Circular Dichroism Spectra analysis. **(A)** CD spectra of KP-PGQs in the presence of 50 mM K^+^, Na^+^, or Li^+^. **(B)** CD spectra of KP-PGQs obtained with the increasing concentration of K^+^ ion.

Circular Dichroism spectroscopy provides an exceptional platform to analyze the folding patterns of macromolecules, especially nucleic acids. CD spectra in the range of 220–320 nm have been extensively explored for analyzing the topologies of different G4 structures where different bands are observed for its different topologies (Vorlíčková et al., 2012; del Villar-Guerra et al., 2018). A negative band at ∼240 nm and a positive band at ∼260 nm depicts the formation of parallel G4, while a negative band at ∼260 nm, and a positive band at ∼290 nm results into an anti-parallel G4 conformation. A negative band at ∼240 nm with positive bands at ∼260 and ∼290 nm are the results of hybrid G4 topology (Vorlíčková et al., 2012; del Villar-Guerra et al., 2018). A mixed or hybrid topology may also be observed when both parallel and anti-parallel conformations are present in the sample. Various cations are reported to stabilize the G4 structures by neutralizing the negative charges present at the center of the G-quartets. Thereby, we explored the G4 topologies in the conserved KP-PGQ sequences in the presence of three mono-cations namely K^+^, Na^+^, and Li^+^ using CD spectral analysis (Figure 3A). In the presence of 50 mM K^+^, all the six KP-PGQs were observed to fold into characteristic G4 conformations *in vitro*. KP-PGQ-1 and KP-PGQ-6 depicted the formation of a mixed hybrid G4 conformations where prominently anti-parallel topology was present with a negative band at ∼260 nm and a positive band at ∼295 nm with a small amount of parallel conformation giving a negative band at ∼240 nm and a small positive bulge at ∼255 nm. This result might occur due to the formation of both parallel and anti-parallel conformations in the solution. KP-PGQ-2 and KP-PGQ-4 were observed to form mixed/hybrid topology with a negative band at ∼240 nm and two positive bands at ∼260 and ∼290 nm, respectively, while KP-PGQ-3 and KP-PGQ-5 predominantly formed parallel topology. We also checked the effect of other physiologically relevant cations—Na^+^ and Li^+^. In the presence of 50 mM Na^+^ and Li^+^, KP-PGQ-1, KP-PGQ-2, KP-PGQ-3, KP-PGQ-4, and KP-PGQ-5 formed parallel conformation while mixed or hybrid conformations were observed in case of KP-PGQ-6 (Figure 3A). On comparing the topology and stability of G4 conformation in the presence of various cations, it was observed that the maximum stability was observed in the presence of K^+^ ion, as the CD ellipticity was highest for this cation. As K^+^ ion is the most relevant cation physiologically, we further analyzed the effect of it on the G4 topology and stability by increasing K^+^ concentration in the solution. In all the six KP-PGQs, with the increase in the concentration of K^+^ from 50 to 200 mM, an increase in the CD ellipticity was observed, depicting further stabilization of G4 conformations (Figure 3B). The mixed hybrid conformation in KP-PGQ-4 and KP-PGQ-6 became evident with increasing concentration of K^+^ from 50 mM to 200 nM (Figure 3B).

*Klebsiella pneumoniae* putative G-quadruplex sequences topologies were also analyzed in the absence of any cations where KP-PGQ-1 formed a parallel topology with a negative band at ∼235 nm and a positive band at ∼255 nm. KP-PGQ-2, KP-PGQ-5, and KP-PGQ-6 formed a mixed topology with a predominance of a parallel conformation while KP-PGQ-3 and KP-PGQ-4 formed parallel conformation in the absence of cations (Figure 3B). On comparative analysis, though KP-PGQs formed G4 conformations even in the absence of any cation, the presence of cations especially K^+^ leads to formation of more stable G4s as depicted by the higher CD ellipticity in the spectra (Figure 3).

**FIGURE 4:**
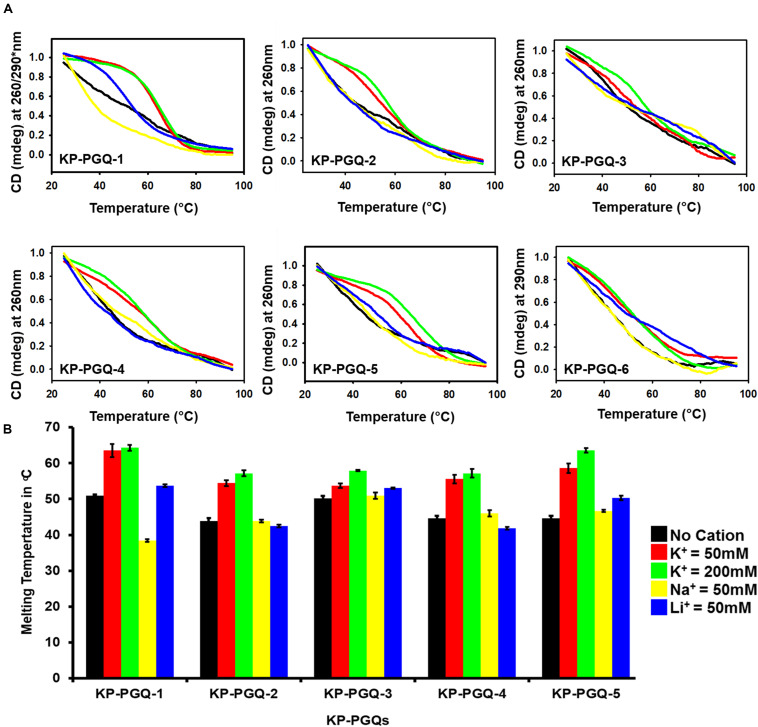
Circular Dichroism melting analysis. **(A)** Thermal denaturation curve of KP-PGQs in the presence and absence of cations obtained by Circular Dichroism (for *T*_*m*_ values please see Supplementary Table S6). **(B)** Comparative analysis of *T*_*m*_ of KP-PGQs in the presence and absence of cations depicted in the form of bar graph. [*For KP-PGQ-1, 260 nm wavelength was used for Na^+^ and Li^+^, while 290 nm was used for K^+^ cation].

To further check the effect of cations on the formation and stabilization of KP-PGQs, CD melting analysis was performed where the KP-PGQs were thermally denatured from 25 to 98°C at the wavelength of maximum CD ellipticity for respective KP-PGQs (Figure 4). For all the KP-PGQ sequences, the highest melting temperature (*T*_*m*_) was observed in case of K^+^ ions, as compared to other cations depicting the highest stabilization in the presence of this cation (Figure 4 and Supplementary Table S8). Thus, maximum CD ellipticity and *T*_*m*_ concluded that K^+^ provides the maximum stabilization of G4 as compared to Na^+^ and Li^+^ ions.

Electrophoretic mobility shift assay, NMR, and CD Spectroscopy confirmed the formation of stable intramolecular G4s in the KP-PGQs *in vitro* under physiological conditions. Given that stable intramolecular G4s act as *cis*-regulators and are able to regulate the gene expression (Parrotta et al., 2014), the conserved KP-PGQs that we observed to present in the promoter region of the essential genes of *K. pneumoniae* may act as potential therapeutic targets against this bacterial infection.

### G4 Selective Ligand BRACO-19 Interacts and Stabilizes the G4 Structures of *Klebsiella pneumoniae*

**FIGURE 5:**
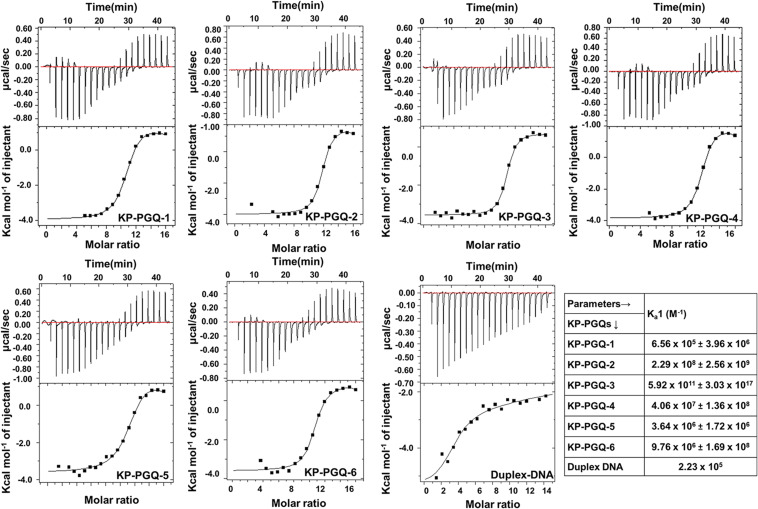
Isotherms obtained from Isothermal Calorimetric analysis for the interaction of BRACO-19 with KP-PGQs and duplex DNA (control) along with their respective association constant (K_*a*_1).

Isothermal calorimetry is one of a prominent technique to analyze the thermodynamic interaction between nucleic acid and ligands. BRACO-19, is one well-known G4 binding ligand and has been shown to possess anti-proliferating and anti-viral effects by influencing the expression of genes harboring G4 motifs in their promoter or coding regions. But no such effect has been reported against bacterial pathogens (Burger et al., 2005; Ruggiero and Richter, 2018). Thus, aiming BRACO-19 as a lead therapeutic compound against *K. pneumoniae* infection via targeting G4, its affinity toward KP-PGQs was observed using isothermal calorimetry (Figure 5). In thermodynamic reactions, negative change in the free Gibbs energy and change in enthalpy (ΔH) depicts the formation of energetically favorable reactions between two molecules. Interestingly, ΔH_1_ for KP-PGQ1, KP-PGQ-2, KP-PGQ-3, KP-PGQ-4, and KP-PGQ-6 was observed to be -3.966 × 10^3^, -1.002 × 10^3^, -3.575 × 10^3^, -3.848 × 10^3^, -3.622 × 10^3^, and -5.419 × 10^3^, respectively, indicating the thermodynamically favored interactions with BRACO-19 and that they are enthalpy driven. ΔH_1_ for KP-PGQ-5 with BRACO-19 was 8.45 × 10^2^, but ΔG_1_ was observed to be -9.525 × 10^3^, showing the feasibility of the reaction and that it is entropically driven. The reactions of KP-PGQs with BRACO-19 were biphasic where the reactions were mostly exothermic and later became endothermic, but the ΔG_1_ and ΔG_2_ were negative in both the cases (Supplementary Table S9). In summary, ITC data analysis with KP-PGQs as ΔG was found to be negative for all KP-PGQs and BRACO-19 complexes supporting the biologically feasible spontaneous and exergonic reactions (Supplementary Table S9). The association constant (K_*a*_1) was highest for KP-PGQ-3 with a value of 5.92 × 10^11^ M^–1^ with ΔH_1_ = -3.575 kcal/mol and ΔS_1_ = 41.9 cal/mol/deg giving ΔG_1_ = -16 kJ which was 10^6^ times higher than the duplex DNA that does not folds into G4 (K_*a*_1 = 2.23 × 10^5^). This result was in line with the fact that BRACO-19 recognizes the parallel G4 conformation with higher affinity, as compared to that of anti-parallel and hybrid/mixed conformation, and CD spectra analysis depicted the parallel conformation of the KP-PGQ-3 in the presence of K^+^ ion (Machireddy and Sullivan, 2019). Interestingly, BRACO-19 showed ∼1,026-, ∼182-, ∼43-, ∼ 16-, and 3-fold higher binding with KP-PGQ-2, KP-PGQ-4, KP-PGQ-6, KP-PGQ-5, and KP-PGQ-1, respectively, as compared to that of duplex DNA (Supplementary Table S9). Thus, ITC analysis showed that BRACO-19 interacts with higher affinity to the KP-PGQs and is spontaneous energetically favored reactions.

**FIGURE 6:**
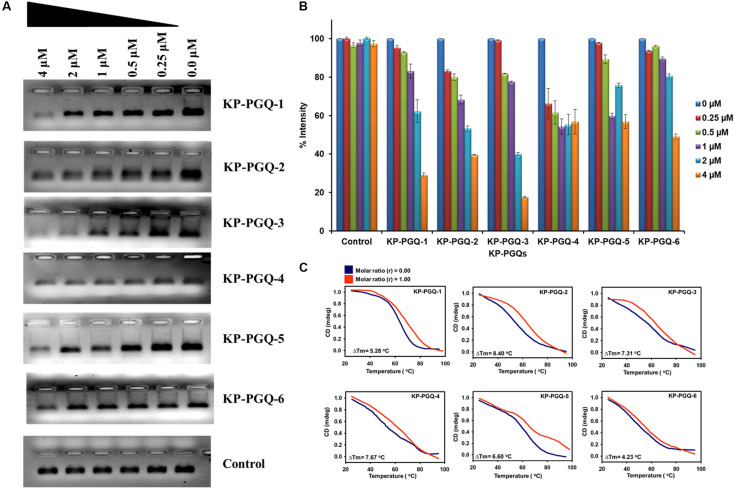
PCR primer extension assays and CD melting analysis of KP-PGQs in the presence of BRACO-19. **(A)** With the increase in BRACO-19 concentration, decrease in the band intensities was observed while no difference was observed for control. **(B)** Comparative analysis of change in band intensities with the change in BRACO-19 concentration and **(C)** CD melting curves with the change in melting temperature (Δ*T*_*m*_) for KP-PGQs in the absence (D/N = 0) and presence of BRACO-19 (D/N = 1).

Additionally, to test the ability of BRACO-19 on folding and stabilization of G4 structure in the conserved PGQs, primer extension and CD melting assay were performed. Formation of G4 in the DNA template acts as a blockage during primer extension and halts the movement of *Taq* polymerase, leading to decrease in the amplification of the PCR product. This change in amplification can be visualized in agarose gel. The PCR reactions were carried out with the DNA templates that consist of KP-PGQ dissolved in 50 mM KCl and a reverse complimentary primer in the presence or absence of BRACO-19. Gel image analysis of the PCR product showed that the increase in the concentration of G4 ligand leads to decreases in the band intensities, while no changes were seen in case of a double-stranded DNA control supporting the folding of G4 motifs in the KP-PGQ DNA templates that arrested the *Taq* polymerase movement (Figures 6A,B). The inhibition of KP-PGQs amplification in the presence of BRACO-19, while there was no significant change in the control sequence, depicted the selective binding of the ligand with the G4 motifs. Maximum inhibition was observed in case of KP-PGQ-3 where ∼80% diminishment in the band intensity was observed at 4 μM BRACO-19 (Figure 6B). This result supported the ITC results that showed the maximum binding affinity of KP-PGQ-3 with BRACO-19. Primer extension assay on KP-PGQs strengthen that the formation of G4 structures can impede biological phenomenon, and thus, these KP-PGQs can play significant role physiologically. As these KP-PGQs are present in the upstream region of the genes of *K. pneumoniae*, similar to primer extension assay observations, the formation of G4s may halt the DNA and RNA polymerases during replication, transcription, and translation *in vivo*, leading to the down-expression of the G4 motif-harboring genes, resulting in bacterial growth inhibition. Furthermore, the stabilization effect of BRACO-19 on KP-PGQs was elucidated using CD melting analysis in the presence and absence of the ligand. The increase in *T*_*m*_ with the addition of BRACO-19 (D/N = 1.0) further confirmed the stabilization of KP-PGQs by the G4 ligand (Figure 6C and Supplementary Table S10).

### Exposure of BRACO-19 Decreases the Expression of *Klebsiella pneumoniae* Genes Harboring G4 Motif in Their Promoter Region

**FIGURE 7:**
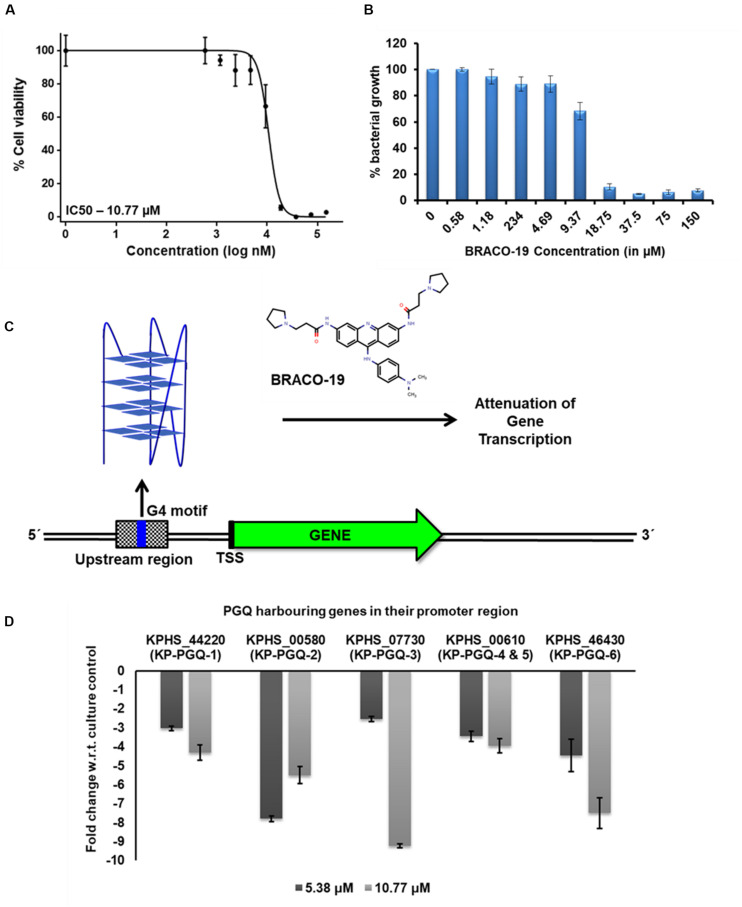
Gene expression analysis. **(A)** Bacterial growth inhibition curve obtained by MTT assay for Klebsiella pneumoniae treated with BRACO-19. **(B)** Bargraph for the MTT assay depicting the bacterial growth (in %) in the presence of different concentrations of BRACO-19. **(C)** Schematic representation of the inhibition of genes harboring G4 motifs in the promoter region. Stabilization of G4 conformation in the presence of BRACO-19 arrests the transcription machinery leading to attenuation of transcription. **(D)** Bar graph depicting the change in gene expression on the addition of BRACO-19 with respect to the culture control conditions. The error bars represent the standard error.

Isothermal calorimetry and primer extension assay revealed the selective binding of BRACO-19 to the KP-PGQs. Furthermore, to analyze the effect of BRACO-19 on the transcription of the *K. pneumoniae* genes harboring G4 motifs in their promoter region, quantitative real-time PCR (qRT-PCR) was performed. Along with high-affinity and specificity for G4s, BRACO-19 has also been reported to be lethal in various pathogens. IC_50_ of BRACO-19 was reported to be in a range of ∼9–12 μM for *Leishmania major* and *P. falciparum* (Belmonte-Reche et al., 2018). In the MTT-based growth inhibition assay, BRACO-19 was able to inhibit the growth of *Klebsiella puemoniae* ATCC 700603 strain with an IC_50_ of 10.77 μM (Figures 7A,B). The presence of these genes harboring G4 motifs in *Klebsiella puemoniae* ATCC 700603 strain was confirmed by using PCR amplification. The stabilization of G4 structures at the G4 motifs by the addition of BRACO-19 could impede the movement of transcriptional machinery, thereby attenuating the transcript formation (Figure 7C). For quantification of gene expression, *Klebsiella puemoniae* ATCC 700603 culture was exposed to two different concentrations of BRACO-19 (5.38 μM and 10.77 μM) for 80 min, and total RNA was isolated. 16S rRNA was used as a housekeeping reference gene, and change in expression of the five genes harboring six conserved KP-PGQs was observed on treating the cultures with the G4 ligand. At both the concentrations of BRACO-19, expression of all the five genes harboring G4 in their promoter region was decreased in comparison to the control conditions depicting the negative role of the G4 motifs in the gene expression. At 5.38 μM BRACO-19, expression of alcohol dehydrogenase (KPHS_00580) harboring KP-PGQ-2 was maximally affected with a fold change of approximately -7.8, while a minimal effect was observed in case of L-ribulose-5-phosphate 4-epimerase (KPHS_07730) gene-harboring KP-PGQ-3, with a fold change of approximately -2.54 (Figure 7D). On increasing the concentration of BRACO-19, the KP-PGQ motif-containing genes were further down-expressed except for KPHS_00580 harboring KP-PGQ-2, which was moderately increased in comparison to the 5.38 μM-treated culture. In 10.77 μM-treated samples, as compared to the untreated conditions, the most under-expressed genes were L-ribulose-5-phosphate 4-epimerase (KPHS_07730), having KP-PGQ-3 (fold change, approx. -9.21; Figure 7D). Increase in the G4 ligand concentration resulted into a moderate changes in expression of genes harboring KP-PGQ-1, KP-PGQ-4, and KP-PGQ-5. The results of quantitative PCR support the hypothesis of the interaction of G4 ligand with these KP-PGQs structures *in vivo*. Therefore, stabilization of the G-quadruples structures could play a critical role in the regulation of the KP-PGQ harboring genes by inhibiting the transcription machinery at the upstream promoter sites. The above observation further supports the essentiality of G4 in gene expression regulation and thus provides a new dimension to the therapeutic approaches for fighting against this notorious multi-drug resistant bacterium.

## Discussion

The presence of highly conserved G4 motifs in the pathogenic bacteria and viral genomes provides a novel platform for designing anti-bacterial and anti-viral therapeutics where these G4s can be used as therapeutic targets against the pathogenic infection and virulence. Evolutionary conservation of these G4 motifs at the genus level shows their critical role in bacterial growth and survival and thus can be used as potential therapeutic targets against both drug-sensitive and drug-resistant bacterial strains.

In this study, we screened the completely sequenced 387 genomes of *K. pneumoniae* to identify the potential G4 motifs that are highly conserved in the *Klebsiella* genus. In bacteria, bidirectional transcription occurs as genes are located in both the strand; hence, both sense and antisense strands were screened for G4 motifs by using an *in-house* developed G4 prediction tool. *In silico* analysis revealed 21 G4 motifs that were conserved in more than 90% of the bacterial strains, out of which six were present at the upstream promoter region of five essential genes. Mapping the G4 motifs in the genome of *K. pneumoniae* revealed the presence of KP-PGQ-1 at the upstream of D-erythrose 4-phosphate dehydrogenase (KPHS_44220), which converts D-erythrose 4-phosphate to 4-phosphoerythronate by utilizing the NAD^+^ molecule, the first step of pyridoxine 5’-phosphate biosynthesis pathway, whose end product, Vitamin B6, is an essential requirement for bacterial growth. KP-PGQ-2 was present at the promoter region of alcohol dehydrogenase (KPHS_00580), which belongs to the carbohydrate metabolism pathway. KP-PGQ-3 was located at the upstream of L-ribulose-5-phosphate 4-epimerase (KPHS_07730) which is involved in L-arabinose metabolism. Two of these highly conserved G4 motifs (KP-PGQ-5 and KP-PGQ-4) were present at the upstream of ABC transporter gene, which plays a significant role in nutrient uptake from the environment to the cytoplasm. KP-PGQ-6 was upstream to 2,4-dienoyl-CoA reductase (KPHS_46430) and is involved in vitamin B6 and fatty acid metabolism. Thus, stabilization of these G4 structures at the promoter regions may influence nutrient transport and various important metabolic pathways and thus can be crucial for bacterial growth. NMR and CD spectroscopic analysis confirmed the formation of G4 structures by all the six conserved motifs, and EMSA analysis depicted their intramolecular nature. The formation of G4 structures by the predicted G4 motif sequences showed the accuracy of the *in-house* prediction tool.

Several ligands like TMPyP4, RPHS, telomestatin, PDS, BRACO-19, and acridine derivatives, etc., have been shown to specifically interact and stabilize the G4 conformations, either by neutralizing the negative charge present on the G4 surface, hydrogen bond formation, or by pi–pi interaction (Ruggiero and Richter, 2018; Asamitsu et al., 2019). Herein, we checked the interaction of one of the most widely used, commercially available G4 ligands, BRACO-19, with conserved KP-PGQs. Isothermal calorimetric analysis showed that BRACO-19 specifically interacts with the G4 structures, and the reactions are thermodynamically favorable. Additionally, the primer extension assay supported the increased stabilization of the KP-PGQs with an increase in the G4 ligand concentration. On comparative thermal denaturation analysis of KP-PGQs in the absence and presence of BRACO-19, the significant increase in the T_*m*_ can be correlated with the greater stabilization of G4 motifs, as more heat was required for unfolding the G4 conformation. In addition to G4 stabilization, the G4 ligand was able to inhibit the bacterial growth in the micromolar range that was significantly lower than the inhibitory concentration of BRACO-19 against the eukaryotic cells (Perrone et al., 2014). Previous research on the role of G4s in the promoter regions of the genes have shown that they act as *cis*-regulating motifs and are negative or positive transcriptional regulators (Perrone et al., 2013; Biswas et al., 2017, 2018). As the KP-PGQs formed the G4 structures *in vitro* and are stabilized by BRACO-19 at physiological conditions, it became imperative to analyze the biological effect of the stabilization of these secondary structures on the expression of the G4 harboring genes. Upon exposure of the bacterial culture with BRACO-19, a significant decrease in the expression of five essential genes harboring the G4 motifs in their promoter region was observed and convincingly strengthens the role of these evolutionary conserved KP-PGQs in negatively regulating the transcription process.

Collectively, this work provides an in depth understanding of evolutionary conserved G4 motifs in *K. pneumoniae* genome. *In silico* analysis revealed the present of G4 motifs in the promoter regions of essential genes involved in metabolism. Various biophysical and biochemical analysis affirmed the formation of G4 conformation by these G4 motifs that may regulate the transcription and translation of the harboring genes. Thus, the present study provides an emerging platform for designing anti-bacterial therapeutics against this nosocomial pathogen.

## Conclusion

In recent times, G4s have emerged as potential therapeutic targets against various human pathogens, including viruses, bacteria, and protozoans. *K. pneumoniae* has become a large threat to humankind, due to the evolution of multi-drug and extensively resistant hypervirulent strains. Therefore, identification of an evolutionarily conserved therapeutic target can help in targeting this infectious pathogen to a greater extends. Herein, we screened the *K. pneumoniae* genomes to identify the conserved putative G4 motifs and used an array of biophysical assay to check the G4 formation *in vitro*. Selective interaction between well-known G4-binding ligand BRACO-19 and KP-PGQs were shown to halt the PCR primer extension process, which highlights its application in gene regulation of KP-PGQs harboring genes. Intriguingly, the interaction between BRACO-19 and KP-PGQ also has been shown to regulate the expression KP-PGQ-harboring genes and inhibited the bacterial growth indicating their critical role in the bacterial survival. In summary, this study showed the occurrence and importance of G4 formation in *K. pneumoniae* genome and their plausible role as potential therapeutic targets against the notorious pathogens.

## Data Availability Statement

All datasets generated for this study are included in the article/Supplementary Material.

## Author Contributions

AK did the data conceptualization and designed the methodology. US and NJ performed the in silico and in vitro experiments. collectively wrote the manuscript. SM, TS, and AK did the critical review and editing. All authors contributed to the article and approved the submitted version.

## Conflict of Interest

The authors declare that the research was conducted in the absence of any commercial or financial relationships that could be construed as a potential conflict of interest.

## Footnotes

1 http://www.ncbi.nlm.nih.gov/genomes

2 http://bioinformatics.ibp.cz:8888/#/analyse/g4-killer
