# Supplementary material for: Conserved G-Quadruplex Motifs in Gene Promoter Region Reveals a Novel Therapeutic Approach to Target Multi-Drug Resistance Klebsiella pneumoniae
Source: Front Microbiol. 2020 Jun 26;11:1269. doi: 10.3389/fmicb.2020.01269 (PMC7344255; doi:10.3389/fmicb.2020.01269)
Supplement: Supplementary file 1 [file Data_Sheet_1.pdf]

Supplementary Material

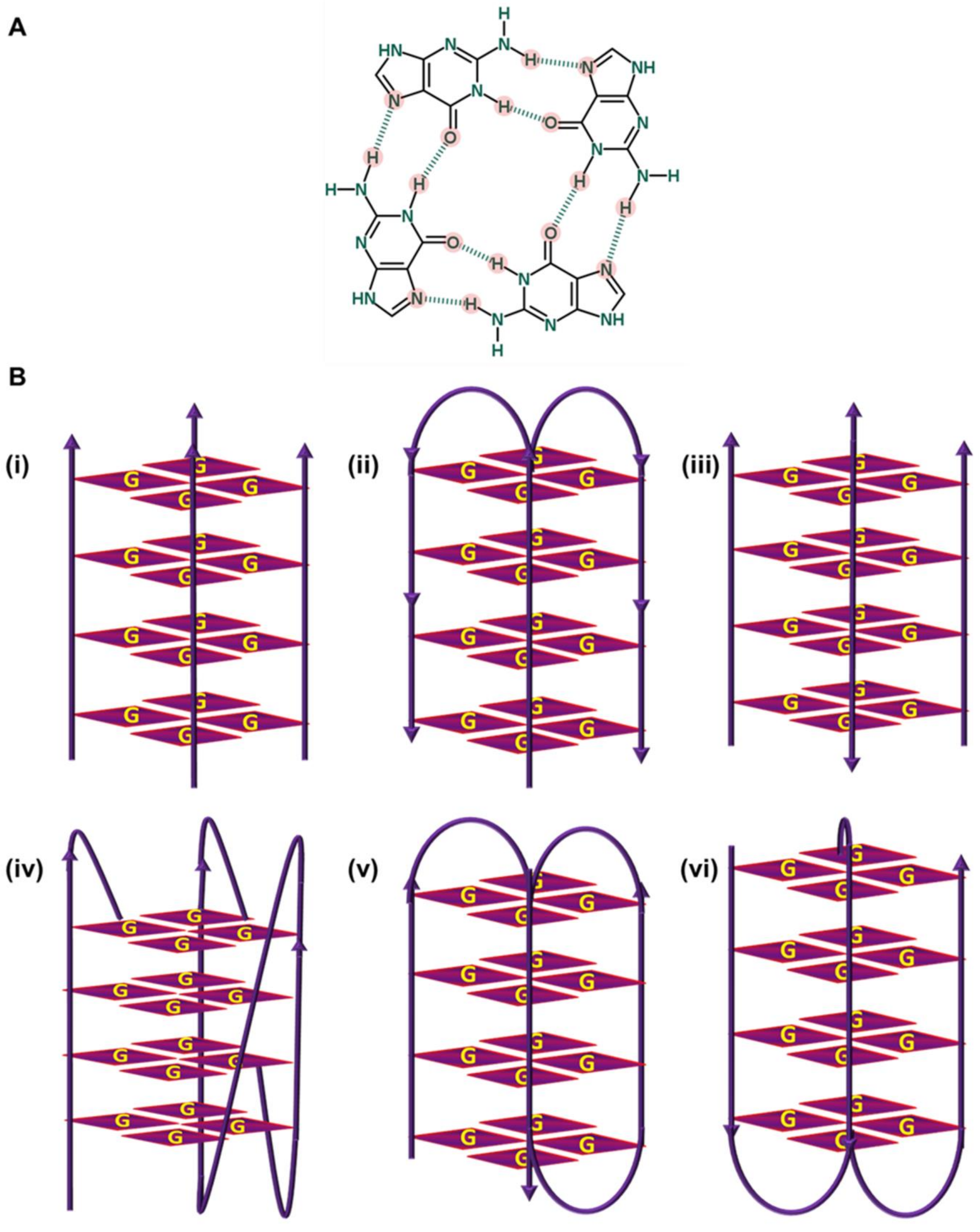

Supplementary Figure S1. **G-quadruplex topologies (A)** G-quartet formed by hoogsteen hydrogen bonds between four guanine residues of same or multiple strands. **(B)** Various topologies adopted by G-quadruplex motifs. (i-iii) Intermolecular G-quadruplexes and (iv-vi) intramolecular G-quadruplex conformation with parallel, antiparallel and hybrid topology respectively.

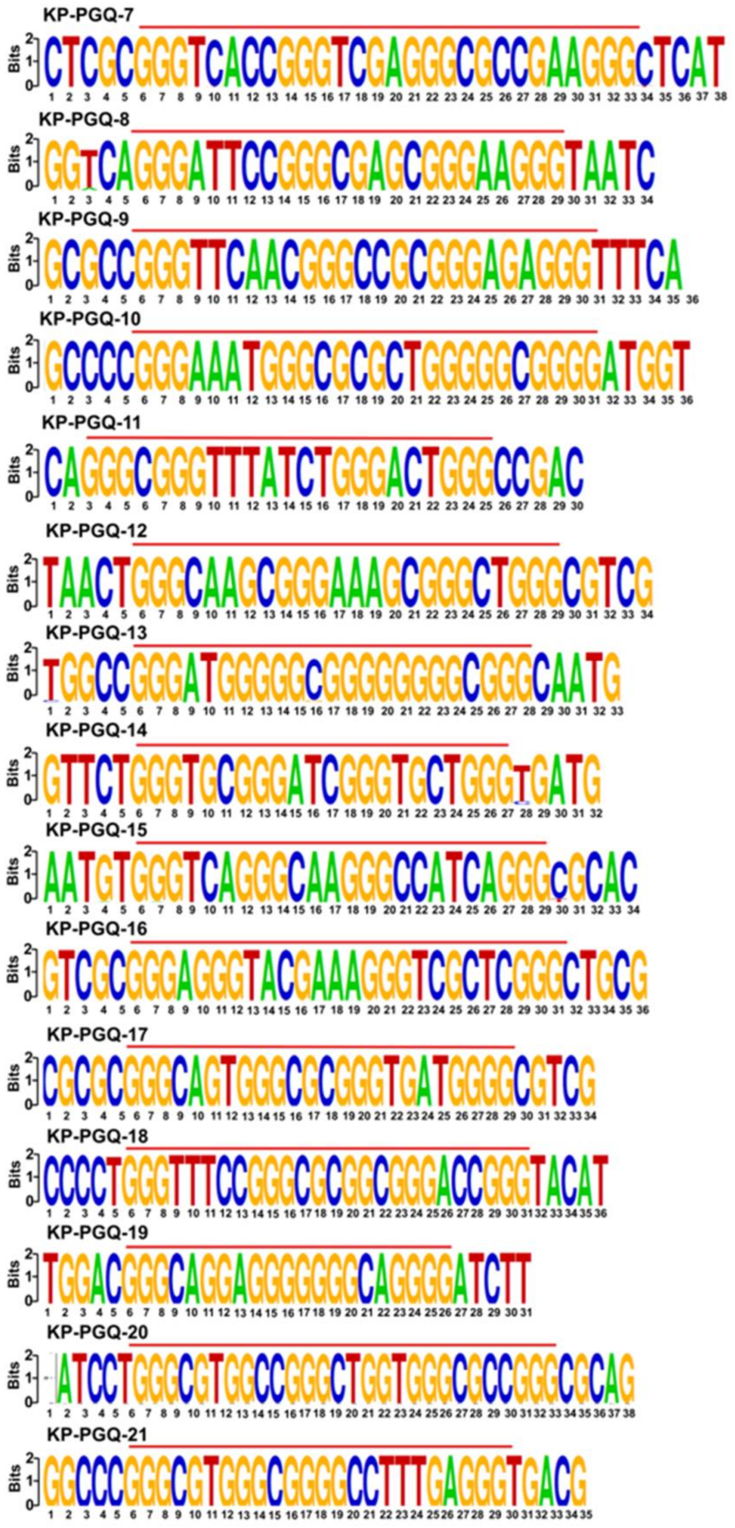

Supplementary Figure S2. Consensus logo for the conserved G-quadruplex motifs that are present in the open reading frame of various genes in *Klebsiella pneumoniae* (KP-PGQ-7 to 21) developed using WebLogo v2 tool.

Supplementary Table S1. List of Oligonucleotide Sequences harboring KP-PGQ motifs used for CD, NMR and ITC analysis (Guanines in the G4 motifs are colored red).

KP_PGQs	Sequence	Length
KP-PGQ-1	CCTGTGGGAGAGGGTTGGGGTGAGGGCATCAG	32
KP-PGQ-2	CCTTTGGGAGAGGGCCGGGGTGTGGGGCAACG	32
KP-PGQ-3	CCAACGGGGAGAGGGGAAGGGTGAGGGGACAGC	33
KP-PGQ-4	CCTGTGGGGGAGGGTTAGGGTGAGGGGTGATT	32
KP-PGQ-5	CCTGCGGGAGAGGGTCGGGGTGAGGGGAAACG	32
KP-PGQ-6	CCTCCGGGAGAGGGCCGGGGTGAGGGAACAA	31

Supplementary Table S2. List of KP-PGQ Mutants used in Electrophoretic mobility shift assay (mutations done in the G4 motifs are colored red)

KP-PGQs	G4 motif	G4Killer_Score (Tool Threshold = 1.2)
**KP-PGQ-1_mutant**	**CCTTTTTTATATTTTTTTTTTTTATTTCATCAT**	-0.188 The target score 1.2 is below the G4Hunter score of the sequence. **No mutation is needed!**
**KP-PGQ-2_mutant**	**CCTTTTTTATATTTCCTTTTTTTTTTTCAACT**	-0.313 The target score 1.2 is below the G4Hunter score of the sequence. **No mutation is needed!**
**KP-PGQ-3_mutant**	**CCAACTTTTATATTTTAATTTTTATTTTACATC**	-0.212 The target score 1.2 is below the G4Hunter score of the sequence. **No mutation is needed!**
**KP-PGQ-4_mutant**	**CCTTTTTTTTATTTTTATTTTTATTTTTTATT**	-0.125 The target score 1.2 is below the G4Hunter score of the sequence. **No mutation is needed!**
**KP-PGQ-5_mutant**	**CCTTCTTTATATTTTCTTTTTTATTTTAAACT**	-0.219 The target score 1.2 is below the G4Hunter score of the sequence. **No mutation is needed!**
**KP-PGQ-6_mutant**	**CCTCCTTTATATTTCCTTTTTTATTTAACAA**	-0.419 The target score 1.2 is below the G4Hunter score of the sequence. **No mutation is needed!**
***Ckit21* mutant**	**TTTCTTTCTCTCTTTATTTT**	-0.2 The target score 1.2 is below the G4Hunter score of the sequence. **No mutation is needed!**

Supplementary Table S3. List of primers used in PCR primer extension assay

KP-PGQs	Sequence (5' → 3')
KP-PGQ-1	TGTGGGAGAGGGTTGGGGTGAGGGGATAGATGGTGAGCAAGGGCGAGGA
KP-PGQ-2	TTTGGGAGAGGGCCGGGGTGTGGGGGATAGATGGTGAGCAAGGGCGAGGA
KP-PGQ-3	AACGGGGAGAGGGGAAGGGTGAGGGGGATAGATGGTGAGCAAGGGCGAGGA
KP-PGQ-4	TGTGGGGGAGGGTTAGGGTGAGGGGGATAGATGGTGAGCAAGGGCGAGGA
KP-PGQ-5	TGCGGGAGAGGGTCGGGGTGAGGGGGATAGATGGTGAGCAAGGGCGAGGA
KP-PGQ-6	TCCGGGAGAGGGCCGGGGTGAGGGGATAGATGGTGAGCAAGGGCGAGGA
Control	AACTTTTATATTTTAATTTTTATTTTTATAGATGGTGAGCAAGGGCGAGGA (G-Killer tool result – 0.353. The target score is below the G4Hunter score of the sequence.)
Reverse Complementary Primer	TCCTCGCCCTTGCTCACCATCT

Supplementary Table S4. List of forward and reverse primers of used for RT-PCR analysis

Gene Name	Primers	Primer Sequence (5' → 3')	Amplicon length
**16S rRNA (Reference)**	**Forward Primer**	**CCCACCTTCCTCCAGTTTATC**	123 bp
	**Reverse Primer**	**GTCAGCTCGTGTTGTGAAATG**	
**KPHS_44220 (KP-PGQ-1)**	**Forward Primer**	**GGCAGGGATAACCCGTATTT**	91 bp
	**Reverse Primer**	**GTCAATTGCCGTCACGTTTATC**	
**KPHS_00580 (KP-PGQ-2)**	**Forward Primer**	**CCCACGCCGACTATTTCTAT**	99 bp
	**Reverse Primer**	**CAATCACCTTGCCGGTTTC**	
**KPHS_07730 (KP-PGQ-3)**	**Forward Primer**	**CCCACGCCGACTATTTCTAT**	99 bp
	**Reverse Primer**	**CAATCACCTTGCCGGTTTC**	
**KPHS_00610 (KP-PGQ-4 & 5)**	**Forward Primer**	**CGTTGTAATCGGTGCTGTAGA**	106 bp
	**Reverse Primer**	**GCTCCTGAGAGAATGATGGATAC**	
**KPHS_46430 (KP-PGQ-6)**	**Forward Primer**	**GATCGGCGGCCAGTTTAATA**	106 bp
	**Reverse Primer**	**CAGATCGACGCCGTTAAGAT**	

Supplementary Table S5: List of Conserved KP-PGQs present in the ORF region of genes in *Klebsiella pneumoniae* genome with their location and functional annotation (Guanines in the G4 motifs are colored red).

KP-PGQs	PG4 motifs	Gene ID	Function	Region in Genome	Gene strand	G4 direction	% Conservedness
KP-PGQ-7	GGGTCACCGGGTCGA GGGCGCCGAAGGG	KPHS_36290	ATPase	ORF region	<<<<	>>>>	97.79
KP-PGQ-8	GGGATTCCGGGCGAG CGGGAAGGG	KPHS_19130	Peroxyureidoacrylate/ureidoacrylate amidohydrolase *rutB*	ORF region	<<<<	>>>>	97.23
KP-PGQ-9	GGGTTCAACGGGCCGC GGGAGAGGG	KPHS_51310	putative lipopolysaccharide heptosyltransferase III	ORF region	>>>>	<<<<	97.23
KP-PGQ-10	GGGAAATGGGCGCGC TGGGGGCGGGG	KPHS_51590	putative voltage-gated ClC-type chloride channel ClcB	ORF region	<<<<	<<<<	96.68
KP-PGQ-11	GGGCGGGTTTATCTGG GACTGGG	KPHS_25270	*lacZ* (beta-galactosidase)	ORF region	<<<<	<<<<	96.13
KP-PGQ-12	GGGCAAGCGGGAAAG CGGGCTGGG	KPHS_07120	bifunctional aspartate kinase/homoserine dehydrogenase I	ORF region	>>>>	<<<<	96.13
KP-PGQ-13	GGGATGGGGGCGGGG GCGGG	KPHS_30990	4-hydroxy-2-oxovalerate aldolase	ORF region	<<<<	<<<<	95.58
KP-PGQ-14	GGGTGCGGGATCGGG TGCTGGG	KPHS_01330	UDP-N-acetylglucosamine 2-epimerase	ORF region	>>>>	>>>>	95.58
KP-PGQ-15	GGGTCAGGGCAAGGG CCATCAGGG	KPHS_22180	putative transport protein	ORF region	>>>>	<<<<	95.58
KP-PGQ-16	GGGAGGGTACGAAAG GGTCGCTCGGG	KPHS_02310	bifunctional isocitrate dehydrogenase kinase/phosphatase	ORF region	>>>>	<<<<	95.58
KP-PGQ-17	GGGCAGTGGGCGCGG GTGATGGGG	KPHS_40750	GntR family transcriptional regulator	ORF region	<<<<	<<<<	95.02
KP-PGQ-18	GGGTTTCCGGGCGCGG CGGGACCGGG	KPHS_43070	NADP(H)-dependent aldo-keto reductase	ORF region	>>>>	<<<<	95.02
KP-PGQ-19	GGGCAGGAGGGGGGG CAGGGG		exodeoxyribonuclease V subunit beta	ORF region			94.47
KP-PGQ-20	GGGCGTGGCCGGGCT GGTGGGCGCCGGG	KPHS_13190	putative ABC transporter	ORF region	>>>.>	>>>>	92.81
KP-PGQ-21	GGGCGTGGGCGGGGC CTTTGAGGG	KPHS_13740	major facilitator superfamily transporter MFS_1	ORF region	<<<<	>>>.	92.26

Supplementary Table S6. G-quadruplex prediction of highly conserved G4 motifs in *Klebsiella pneumoniae* strains using G4Hunter tool (Guanines in the G4 motifs are colored red).

KP-PGQs	G4 motif	G4Hunter_Score (Tool Threshold = 1.2)
KP-PGQ-1	GGGAGAGGGTTGGGGTGAGGG	2.143
KP-PGQ-2	GGGAGAGGGTCGGGGTGAGGGG	2.318
KP-PGQ-3	GGGGAGAGGGGAAGGGTGAGGGG	2.562
KP-PGQ-4	GGGGGAGGGTTAGGGTGAGGGG	2.5
KP-PGQ-5	GGGAGAGGGCCGGGGTGTGGGG	2.182
KP-PGQ-6	GGGAAATGGGCGCGCTGGGGGCGGGG	2
KP-PGQ-7	GGGTCGAGGGCGCCGAAGGG	1.2
KP-PGQ-8	AGGGATTCCGGGCGAGCGGGAAGGGT	1.231
KP-PGQ-9	GGGTTCAACGGGCCGCGGGAGAGGG	1.24
KP-PGQ-10	GGGAAATGGGCGCGCTGGGGGCGGGG	2
KP-PGQ-11	GGGCGGGTTTATCTGGGACTGGG	1.435
KP-PGQ-12	GGGCAAGCGGGAAAGCGGGCTGGG	1.417
KP-PGQ-13	GGGATGGGGGCGGGGGCGGG	2.8
KP-PGQ-14	GGGTGCGGGATCGGGTGCTGGG	1.591
KP-PGQ-15	GAATGTGGGTCAGGGCAAGGGC	1.36
KP-PGQ-16	GGGAGGGTACGAAAGGGTCGCTCGGG	1.308
KP-PGQ-17	GGGCAGTGGGCGCGGGTGATGGGG	1.792
KP-PGQ-18	GGGTTTCCGGGCGCGGCGGGACCGGG	1.25
KP-PGQ-19	GGGCAGGAGGGGGGGCAGGGG	2.619
KP-PGQ-20	GGGCGTGGCCGGGCTGGTGGG	1.429
KP-PGQ-21	GGGCGTGGGCGGGGCCTTTGAGGG	1.625

Supplementary Table S7. G-quadruplex prediction of highly conserved G4 motifs in *Klebsiella pneumoniae* strains using QGRS Mapper tool (Guanines in the G4 motifs are colored red).

KP-PGQs	G4 motif	QGRS Score
KP-PGQ-1	GGGAGAGGGTTGGGGTGAGGG	42
KP-PGQ-2	GGGAGAGGGCCGGGGTGTGGGG	42
KP-PGQ-3	GGGGAGAGGGGAAGGGTGAGGGG	42
KP-PGQ-4	GGGGGAGGGTTAGGGTGAGGGG	42
KP-PGQ-5	GGGAGAGGGTCGGGGTGAGGGG	42
KP-PGQ-6	GGGAAATGGGCGCGCTGGGGGCGGGG	40
KP-PGQ-7	GGGTCACCGGGTCGAGGGCGCCGAAGGG	39
KP-PGQ-8	GGGATTCCGGGCGAGCGGGAAGGG	39
KP-PGQ-9	GGGTTCAACGGGCCGCGGGAGAGGG	39
KP-PGQ-10	GGGAAATGGGCGCGCTGGGGGCGGGG	40
KP-PGQ-11	GGGCGGGTTTATCTGGGACTGGG	36
KP-PGQ-12	GGGCAAGCGGGAAAGCGGGCTGGG	39
KP-PGQ-13	GGGATGGGGGCGGGGGCGGG	41
KP-PGQ-14	GGGTGCGGGATCGGGTGCTGGG	41
KP-PGQ-15	GGGTCAGGGCAAGGGCCATCAGGG	39
KP-PGQ-16	GGGAGGGTACGAAAGGGTCGCTCGGG	36
KP-PGQ-17	GGGCAGTGGGCGCGGGTGATGGGG	41
KP-PGQ-18	GGGTTTCCGGGCGCGGCGGGACCGGG	39
KP-PGQ-19	GGGCAGGAGGGGGGGCAGGGG	38
KP-PGQ-20	GGGCGTGGCCGGGCTGGTGGGCGCCGGG	39
KP-PGQ-21	GGGCGTGGGCGGGGCCTTTGAGGG	37

Supplementary Table S8. Melting temperature (T_m) in °C of KP-PGQs obtained in the absence and presence of various cations in Circular Dichroism melting assay.

T_m in the presence of Cations (in °C)→ / KP-PGQs ↓	No Cation	K^+= 50 mM	K^+= 200 mM	Na^+= 50mM	Li^+= 50mM
KP-PGQ-1	50.937	63.57	64.29	38.39	53.71
KP-PGQ-2	43.88	54.43	57.17	43.89	42.47
KP-PGQ-3	50.2	53.75	57.94	50.93	53.04
KP-PGQ-4	44.6	55.56	57.2	45.99	41.83
KP-PGQ-5	44.67	58.61	63.58	46.7	50.28
KP-PGQ-6	43.24	50.89	51.6	43.14	48.81

Supplementary Table S9. Thermodynamic parameters obtained from the interaction of KP-PGQs with BRACO-19 by fitting ITC data in two mode binding model.

Parameters → / KP-PGQs ↓	K_a1 (M^{-1})	ΔH_1 (cal/mol)	ΔS_1 (cal/mol/deg)	ΔG_1 (Joules)	ΔK_a2 (M^{-1})	ΔH_2 (cal/mol)	ΔS_2 (cal/mol/deg)	ΔG_2 (Joules)
KP-PGQ-1	$6.56 \times 10^5 \pm 3.96 \times 10^6$	$-3.966 \times 10^3 \pm 47.4$	13.3	-7.929×10^3	$3.81 \times 10^3 \pm 3.38 \times 10^4$	$7.805 \times 10^4 \pm 1.72 \times 10^7$	278	-4.794×10^3
KP-PGQ-2	$2.29 \times 10^8 \pm 2.56 \times 10^9$	$-1.002 \times 10^3 \pm 107$	24.8	-8.392×10^3	$1.83 \times 10^6 \pm 2.15 \times 10^7$	$1.581 \times 10^3 \pm 580$	340	-9.973×10^4
KP-PGQ-3	$5.92 \times 10^{11} \pm 3.03 \times 10^{17}$	$-3.575 \times 10^3 \pm 44.3$	41.9	-1.606×10^4	$1.27 \times 10^9 \pm 1.21 \times 10^{12}$	$7.20 \times 10^2 \pm 71.1$	44.1	-1.242×10^4
KP-PGQ-4	$4.06 \times 10^7 \pm 1.36 \times 10^8$	$-3.848 \times 10^3 \pm 60.5$	21.9	-2.678×10^3	$3.71 \times 10^5 \pm 1.34 \times 10^6$	2.085×10^3	32.5	-7.6×10^3
KP-PGQ-5	$3.64 \times 10^6 \pm 1.72 \times 10^6$	$-3.622 \times 10^3 \pm 96.1$	17.9	-8.956×10^3	1.00×10^5	$2.205 \times 10^3 \pm 8.45 \times 10^2$	30.3	-6.824×10^3
KP-PGQ-6	$9.76 \times 10^6 \pm 1.69 \times 10^8$	$8.450 \times 10^2 \pm 207$	34.8	-9.525×10^3	$8.80 \times 10^8 \pm 1.51 \times 10^{10}$	$-3.606 \times 10^3 \pm 92.3$	28.8	-1.218×10^4
CT-DNA	2.23×10^5	$-5.419 \times 10^3 \pm 648$	6.29	-7.293×10^3	0.982	-2.919×10^8	-9.79×10^5	-1.58×10^5

Supplementary Table S10. Change in Melting temperature (T_m, °C) observed in KP-PGQs on the addition of BRACO-19 at D/N = 1.0 obtained by Circular Dichroism melting assay.

KP-PGQs	T_m (In absence of BRACO19, D/N= 0.0) (in °C)	T_m (in presence of BRACO-19, D/N = 1.0) (in °C)	ΔTm
KP-PGQ-1	63.57	68.85	5.28
KP-PGQ-2	54.43	62.83	8.4
KP-PGQ-3	53.75	61.42	7.67
KP-PGQ-4	55.56	62.87	7.31
KP-PGQ-5	58.61	65.21	6.6
KP-PGQ-6	50.89	55.12	4.23
